# Identification of a Novel ZIC3 Isoform and Mutation Screening in Patients with Heterotaxy and Congenital Heart Disease

**DOI:** 10.1371/journal.pone.0023755

**Published:** 2011-08-17

**Authors:** James E. J. Bedard, Allison M. Haaning, Stephanie M. Ware

**Affiliations:** Department of Pediatrics, Cincinnati Children's Hospital Medical Center and University of Cincinnati College of Medicine, Cincinnati, Ohio, United States of America; Tor Vergata University of Rome, Italy

## Abstract

Patients with heterotaxy have characteristic cardiovascular malformations, abnormal arrangement of their visceral organs, and midline patterning defects that result from abnormal left-right patterning during embryogenesis. Loss of function of the transcription factor ZIC3 causes X-linked heterotaxy and isolated congenital heart malformations and represents one of the few known monogenic causes of congenital heart disease. The birth incidence of heterotaxy-spectrum malformations is significantly higher in males, but our previous work indicated that mutations within ZIC3 did not account for the male over-representation. Therefore, cross species comparative sequence alignment was used to identify a putative novel fourth exon, and the existence of a novel alternatively spliced transcript was confirmed by amplification from murine embryonic RNA and subsequent sequencing. This transcript, termed *Zic3-B*, encompasses exons 1, 2, and 4 whereas *Zic3-A* encompasses exons 1, 2, and 3. The resulting protein isoforms are 466 and 456 amino acid residues respectively, sharing the first 407 residues. Importantly, the last two amino acids in the fifth zinc finger DNA binding domain are altered in the Zic3-B isoform, indicating a potential functional difference that was further evaluated by expression, subcellular localization, and transactivation analyses. The temporo-spatial expression pattern of *Zic3-B* overlaps with *Zic3-A in vivo*, and both isoforms are localized to the nucleus *in vitro*. Both isoforms can transcriptionally activate a Gli binding site reporter, but only ZIC3-A synergistically activates upon co-transfection with Gli3, suggesting that the isoforms are functionally distinct. Screening 109 familial and sporadic male heterotaxy cases did not identify pathogenic mutations in the newly identified fourth exon and larger studies are necessary to establish the importance of the novel isoform in human disease.

## Introduction

Mutations in the gene encoding zinc finger of the cerebellum protein 3 (*ZIC3*; NM_003413.3) result in heterotaxy and can cause isolated congenital heart defects (CHD). Clinical features of heterotaxy, or *situs ambiguus*, include complex CHDs and low survival rates in affected individuals. There is a spectrum of typical cardiac defects in heterotaxy, including dextrocardia, transposition of the great arteries, and double outlet right ventricle, among others [1], [2].

ZIC3, a member of the GLI superfamily of transcription factors, contains five highly conserved C2H2-type zinc finger domains that span over one-third of the protein and are required for DNA-binding and subsequent transcriptional activation [3], [4], [5], [6], [7]. Previously, ZIC3 was shown to bind and activate transcription at a Gli consensus binding sequence [3], [6] as well as a distinct cis-element in the Nanog promoter [7]. The zinc finger DNA binding domains of ZIC3 are important in performing other complex functions as well, such as binding to co-proteins and directing proper cellular trafficking during development [6], [8], [9], [10].

Although ZIC3 is critical for neural, neural crest, mesoderm, and left-right axis development, its precise functions during development and body pattern formation are not known [2], [11], [12], [13], [14], [15]. Previous work from our lab and others has shown that mutations and deletions of *ZIC3-A* result in cardiovascular malformations and visceral situs anomalies [10], [16], [17], [18], [19], [20], [21] due to loss of function. Deletion of *Zic3* in mouse recapitulates the phenotypes found in patients with *ZIC3-A* mutations [2], [21], providing additional evidence for its role in left-right patterning.

It is estimated that up to 74% of all human genes express multiple mRNAs by alternative splicing of their pre-mRNAs [2], [22]. Alternative splicing is an effective mechanism that facilitates an increase in mRNA and protein diversity without increasing overall gene numbers. Here we identify and characterize ZIC3-B, a novel alternative isoform of ZIC3, hereafter called ZIC3-A; compare the abilities of the two isoforms to activate transcription at the Gli binding site, a known target; and screen for mutations within the newly identified alternative exon in patients with heterotaxy and CHD.

## Materials and Methods

### Ethics Statement

The Institutional Animal Care and Use Committee of Cincinnati Children's Hospital approved this study (IACUC protocol number 0C07054). Anonymized human skin fibroblast cells were analyzed. These cells are considered non-human subject research by the Cincinnati Children's Hospital Institutional Review Board. Patient samples were originally collected under an IRB approved protocol from Baylor College of Medicine. De-identified samples were provided for this study and are categorized as non-human subject research. The Cincinnati Children's Hospital Institutional Review Board has approved this study.

### Cell culture conditions and transfections

NIH/3T3 and HeLa cells were obtained from the American Type Culture Collection. All cells were grown in high glucose Dulbecco's Modified Eagle's Medium (Gibco) and supplemented with 10% fetal bovine serum (Gibco). For each experiment, cells were counted using a hemocytometer and plated to achieve 50% density the following day for transfections. Cells were transfected using Lipofectamine and Plus reagents (Invitrogen) or Fugene HD (Roche) for 24–48 hours as per the manufacturer's recommendations.

### RNA isolation, reverse-transcriptase (RT)-PCR, and real-time PCR

RNA was extracted from C57BL/6 mouse tissues and cultured cells using Trizol reagent (Invitrogen) according to the manufacturer's recommendations. RNA was quantified using a Biomate3 spectrophotometer (Thermo Electron Corporation). RT-PCR was performed using the Titan One Tube RT-PCR System (Roche) as per manufacturer's recommendations, using 50 ng of total RNA as template. Amplification of the entire murine *Zic3-A* open reading frame was performed using the following primer pair: mZic3–5′UTR-Forward – 5′-GCC TGC ACC CTT GCT CAC TTC-3′ and mZic3–3′UTR-Reverse – 5′-GGC CTC TTG CTT TAA AAC TCC-3′. Amplification of *Zic3-B* exon 2-4 region was performed using the primer pair: Zic3-Exon2-EcoRI-Forward: 5′-TTT CAA ATG TGA ATT CGA AGG-3′ and Zic3-B-ORF-Reverse: 5′-TCA GTA AAT CAT TTC TTG CAC A-3′. The following *Zic3* oligonucleotide primers were used to differentiate between murine isoforms by gel electrophoresis: Zic3 Forward – 5′-AAG GCT GTG ACA GAC GGT TT-3′; Zic3-A Reverse – 5′-TTG TGG CTG GTG CTA GTT TG-3′; and Zic3-B Reverse – 5′-TTG CTG CAT ACC AAC GTC AG-3′. The same primers were used to amplify isoforms from cultured human cell RNA with the exception of the Zic3-B reverse primer, which was substituted with ZIC3-B Reverse 5′-CTG GCT GCT GCA TAC CAA C-3′. Murine-specific *β-actin* oligonucleotides were: mActin-Forward: 5′-TTC TTT GCA GCT CCT TCG TT-3′ and mActin-Reverse: 5′-CTT CTC CAT GTC GTC CCA GT-3′. Total mRNA was converted into cDNA using the Transcriptor First Strand cDNA Synthesis Kit (Roche) as per manufacturer's recommendations using the provided anchored-oligo (dT)_18_ primer.

### Expression constructs

The mZic3-5′UTR-F-mZic3-3′UTR-R amplicon was TA-cloned into the pGEM-T vector (Promega) and subsequently used as a template for amplification of the *Zic3-A* open reading frame region using the following primers containing *Kpn*I restriction sites: mZic3-A-ORF-Forward – 5′-AAC AAC *GGT ACC* ATG ACG ATG CTC CTG CAC G-3′ and mZic3-A-ORF-Reverse – 5′-TTT TGG *GGT ACC* TCA GAC GTA CCA TTC GTT AAA ATT G-3′. The *Zic3-A* PCR product was subsequently digested with *Eco*RI and *Kpn*I restriction endonucleases and subcloned in-frame into a pFLAG-CMV-2 expression vector (Sigma). Utilizing a unique *Eco*RI restriction site in *Zic3*, the 3′-region of *Zic3-A* was replaced with the digested *Zic3-B* exon2-4 insert to create the entire murine *Zic3-B* ORF. The *Zic3-B* ORF was then subcloned in-frame into a pHM6 vector (Roche) in order to be comparable to previously characterized HA-tagged wild-type and mutant (S43X) ZIC3-A [3]. All expression constructs were verified by DNA sequencing. For dual luciferase assays, a firefly luciferase-expressing 12xGLIBS-luc [23] reporter and a renilla luciferase-expressing pRL-TK reporter (Promega) were used. Flag-GLI1 and Flag-GLI3 constructs were described previously [24], [25].

### Heterotaxy Cohort

Patients were ascertained on the basis of cardiovascular malformations and were categorized as having classic heterotaxy or CHD heterotaxy as described previously [10]. For the current study, only samples from males were screened. Briefly, patients with complex cardiovascular malformations and evidence of disrupted left-right patterning in at least one other organ system were classified as classic heterotaxy. Patients were categorized as having CHD heterotaxy if they had normal situs in other organs and vasculature or if insufficient information was available to determine extra-cardiac organ position. The cardiovascular malformations ascertained were based on those described in the Baltimore-Washington Infant Study. All patients had complex defects with more than one abnormality of cardiac structure or position with the exception of 10 patients that had isolated D-transposition of the great arteries (TGA). Patients with situs inversus totalis were not included. Table 1 summarizes the cardiovascular malformations in the heterotaxy CHD group.

**Table 1:** Cardiovascular Malformations in the CHD Heterotaxy Group.

Classic Heterotaxy	60
CHD Heterotaxy^a^	49
** Cardiac position**	
levocardia	34
mesocardia	2
dextrocardia	13
** Atria**	
Isomerism or inversion	2
ASD	2
AV canal or atresia	3
TAPVR or PAPVR	2
** Ventricles**	
VSD	10
VI	4
Hypoplastic left ventricle	5
MA	3
** Great Arteries**	
D-TGA	29
DORV	3
L-TGA	15
PS or PA	8
AS	1
CoA	9

a Ten patients had isolated D-TGA. The remainder had multiple cardiovascular malformations.

ASD, atrial septal defect; AV, atrioventricular; TAPVR, total anomalous pulmonary venous return; PAPVR, partial anomalous pulmonary venous return; VSD, ventricular septal defect; VI, ventricular inversion; MA, mitral atresia; D-TGA, D-transposition of the great arteries; DORV, double outlet right ventricle; L-TGA, L-transposition of the great arteries; PS, pulmonic stenosis; PA, pulmonic atresia; AS, aortic stenosis; CoA, coarctation of the aorta.

### Mutation screening

All patient samples were analyzed for mutations in the coding region of exon 4 and the splice acceptor site as described below. Thirty of 109 patients had previously been analyzed by full sequencing of ZIC3-A [10]. Patients that were not previously sequenced for ZIC3-A underwent sequencing using primers described previously [10]. Patient genomic DNA was isolated and prepared by standard procedures. Amplification of DNA was performed by PCR in reaction volumes of 20 µL containing the following reagents: 2 µL of 10X Taq polymerase reaction buffer (Fisher), 0.2 U *Taq* polymerase (Fisher), 0.2 nM of each oligonucleotide primer, and 50 ng of genomic DNA template. Oligonucleotide sequences were: *ZIC3*-EXON4-Forward: 5′-AAG AGG AAA TGT GGC CTG TTT-3′ and *ZIC3*-EXON4-Reverse 5′-CAC TTC AAG GTA ACA GAC ATC CA-3′. Amplifications were performed using standard PCR conditions and annealing at 55°C. DNA sequencing was performed on an ABI Prism 3730 sequencer and variants were identified using SeqMan Pro (DNA Star) software followed by manual review.

### Protein isolation, western blotting and immunohistochemistry

Cells were washed in ice-cold PBS before the addition of lysis buffer (3.0 mM EDTA, 1% Triton X-100, 0.1 mM sodium orthovanadate, 1 mM phenylmethylsulfonyl fluoride) with protease (Roche) and phosphatase (Sigma) inhibitors. Whole cell lysate was freeze-thawed and vortexed briefly, followed by centrifugation at 4°C for 10 min to pellet cellular debris. Protein samples were quantified by the method of Lowry et al. [26]. Samples were electrophoresed on 10% Tris-Glycine mini gels (Invitrogen) using the XCell SureLock Mini-Cell (Invitrogen) according to manufacturer's recommendations. Gels were transferred to pre-soaked Immuno-Blot PVDF membranes (Invitrogen) using the XCell II Blot Module (Invitrogen). Western blots were blocked in 5% non-fat dry milk in 1X PBST at room temperature for one hour. Anti-ZIC3 C-terminal (C-13, catalog # SC-28156) and N-terminal (N-19, catalog # SC-28154) polyclonal antibodies were purchased from Santa Cruz Biotechnologies. Following overnight incubation with primary antibodies, blots were washed three times, 10 min each, in 1X PBST, followed by one hour incubation in 5% milk in PBST with the appropriate HRP conjugated secondary antibody. Blots were then washed again three times, 10 min each, in 1X PBST before detection using ECL Plus Detection Reagent (GE Healthcare) and autoradiography film (Denville Scientific). Immunohistochemistry and subcellular localization assays were performed as previously described [4]. The anti-FLAG antibody was purchased from Stratagene (catalog # 200470-21).

### Dual luciferase assay

Flag-GLI3, HA-ZIC3-A, HA-ZIC3-S43X, and HA-Zic3-B constructs were co-transfected as indicated with the reporters 12xGLIBS-luc [23] and pRL-TK (Promega) into NIH/3T3 cells using Fugene HD. Transactivation levels with human HA-ZIC3-A and mouse HA-Zic3-A were previously shown to be identical and HA-ZIC3-A was used for current experiments for comparison with HA-ZIC3-S43X, a known patient mutation. Cell lysates were harvested after 48 hours using Passive Lysis Buffer (Promega). A Dual Luciferase Assay Kit (Promega) and a microplate luminometer (Turner Biosystems) were used to measure luminescence from cell lysates in triplicate, according to the manufacturer's recommendations. Firefly luciferase readings were normalized to renilla luciferase readings, and ratios were normalized to a baseline ratio generated by co-transfection of empty expression vectors with both reporters. Statistical difference between values was determined by unpaired Student's t-test, assuming unequal variance, and differences were considered significant if the resulting probability value was less than 0.05.

## Results

### Prediction of a novel *Zic3* exon

**Figure 1:**
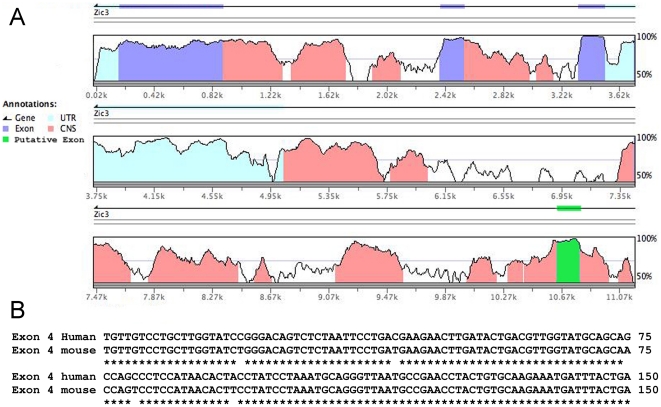
Cross species comparative sequence alignment. **(A)** Genomic alignment across 11 kb of human and murine DNA containing the *Zic3* gene. Known and putative *Zic3* exons, untranslated regions (UTR) and non-coding sequences (CNS) are indicated. All exons are conserved at a minimum 91% identity between human and murine sequences. **(B)** Sequence alignment of the putative exon 4 in human and mouse demonstrates 97% identity.

According to current estimates, approximately 1% of sporadic CHD and the majority of X-linked heterotaxy cases are caused by mutations in ZIC3 in the human population. These results indicate that, although there is a significant overrepresentation of males affected with heterotaxy, it is not due only to mutations in the currently identified form of ZIC3. This raised the possibility that mutations in a previously unrecognized, alternatively spliced form of ZIC3 might contribute to heterotaxy and CHD cases. To investigate this hypothesis, genomic DNA fragments containing *ZIC3* were aligned and compared using mVista software (http://genome.lbl.gov/vista/mvista/submit.shtml) to search for nucleotide homology across species (Figure 1A) [27]. Regions of high homology were also screened for consensus (AG) splice acceptor sites. These analyses revealed a region that is 97% similar in human and mouse (Figure 1B), located approximately 5 kb downstream from the *ZIC3* gene. Further *in silico* analyses confirmed a potentially spliced *ZIC3* mRNA (http://www.ensembl.org – Transcript ID: ENST00000370606) [8], [10]. The predicted spliced form is identical to *ZIC3* except for the last exon, which, if expressed, would result in a protein with a unique carboxy (C)-terminal region. An additional predicted ZIC3 isoform containing a truncation of 220 amino acids from the amino (N)-terminus was also identified *in silico*, but was not further investigated in this current study as it did not encompass a novel, putative exon.

### Murine *Zic3* contains four exons that are alternatively spliced

**Figure 2:**
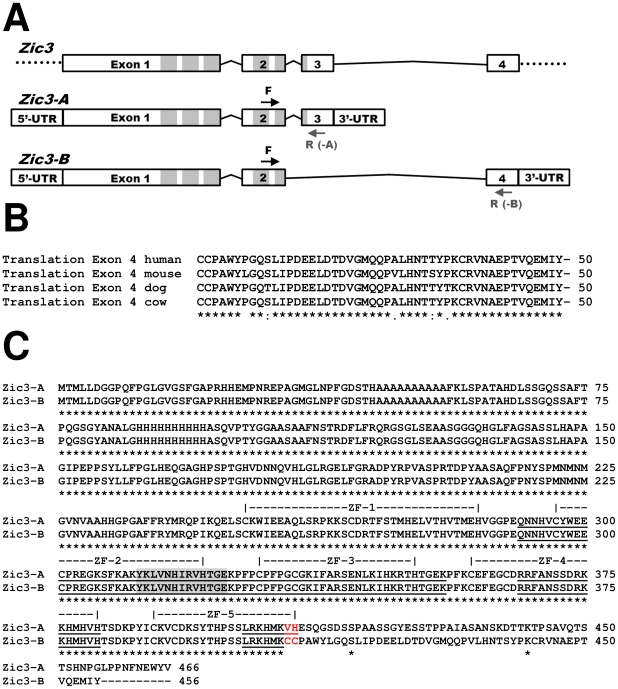
Genomic structure and alternative splicing of *Zic3*. **(A)** Schematic depiction of the genomic structure of *Zic3*. *Zic3-A* consists of exons 1, 2, and 3 while *Zic3-B* consists of exons 1, 2, and 4. Primer sites used for isoform specific amplification in exons 2–3 and exons 2–4 are indicated. F  =  forward, R(-A)  =  reverse for *Zic3-A*, and R(-B)  =  reverse for *Zic3-B* isoforms. **(B)** Sequence alignment of exon 4 translation across species. (*) identical, (:) functionally conserved or (.) semi-conserved amino acid residues are indicated. **(C)** Sequence alignment of murine Zic3-A and Zic3-B protein isoforms. Zinc finger domains (ZF-1–5) are indicated by brackets. NLS regions are underlined and NES region is shaded in gray. The last two amino acids in the fifth zinc finger domain that differ between the two isoforms are indicated in red. ZF, zinc finger.

**Figure 3:**
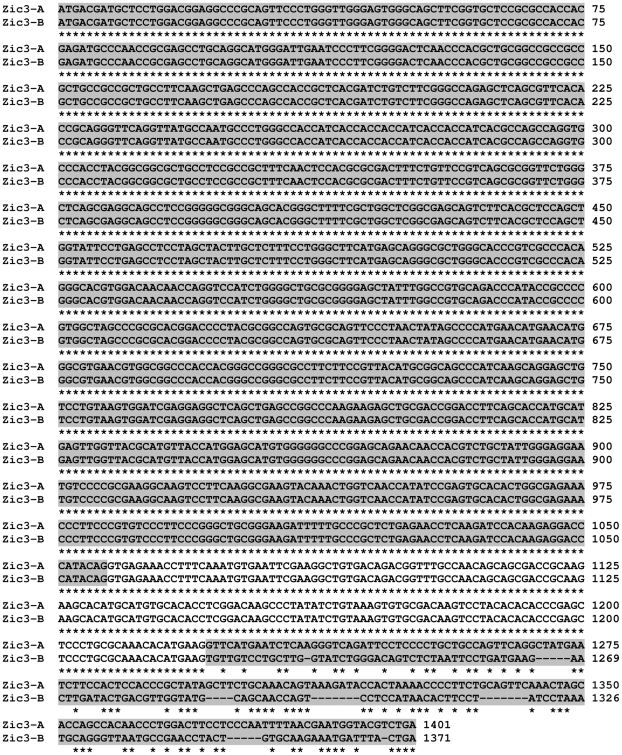
Alignment of the coding sequences of *Zic3-A* and *Zic3-B* transcripts. The coding sequences of murine *Zic3-A* and *Zic3-B* were aligned. Alternating exons are indicated by gray shading. The last exons aligned are exons 3 and 4 for *Zic3-A* and *-B*, respectively, demonstrating less than 35% identity.

The gene structure of *Zic3* is therefore comprised of a total of four exons, of which exons 3 and 4 are alternatively spliced (Figure 2A). Alignment of exons 3 and 4 demonstrates less than 35% identity between them (Figure 3), suggesting that exon 4 evolved independently and did not arise by a duplication and divergence of exon 3.

Cross species conservation is a known feature of exon coding regions. The translation of the novel mRNA sequence and cross-species comparison further demonstrates that the coding region of exon 4 is highly conserved in mammals (Figure 2B). This finding provides additional evidence for the existence of a novel Zic3-B protein isoform. The resulting protein isoforms are 466 and 456 amino acid residues respectively, sharing the first 407 residues. Because of the extensive sharing of residues, many of the important structural domains are identical in Zic3-A and Zic3-B (Figure 2A and 2C). Zic3-A has five zinc finger domains, the first four of which are encoded by exons 1 and 2. The fifth zinc finger domain, encoded by exons 2 and 3, contains a nuclear localization signal (NLS). Interestingly, Zic3-A and Zic3-B differ at the last two amino acid residues of the fifth zinc finger domain (Figure 2C). Because these last two amino acids are in an NLS region [8] and in an alpha-helical region that is essential for DNA binding [28], the function of the fifth zinc finger domain is most likely altered in the Zic3-B isoform. The other two known NLS domains [8], [29] and the nuclear export signal (NES) domain [8] are intact in the Zic3-B isoform (Figure 2C).

### Developmental expression profile of *Zic3-A* and *-B* mRNA

**Figure 4:**
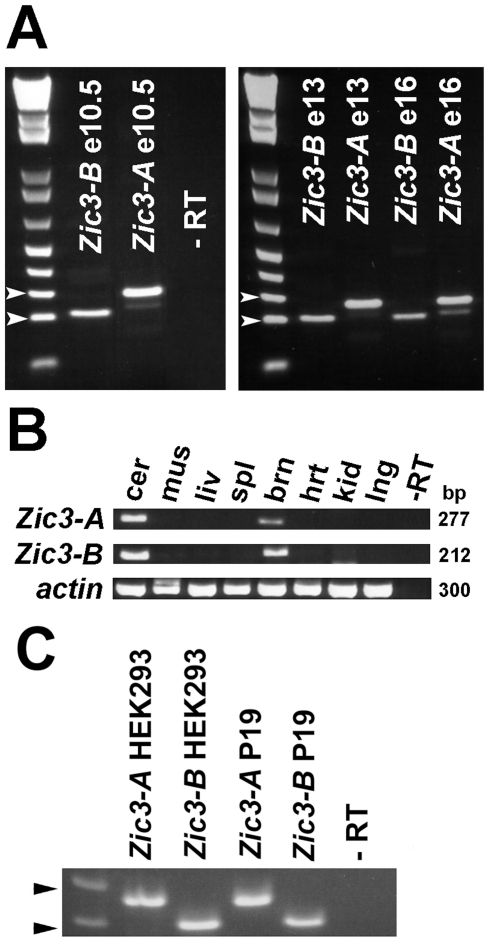
Comparative expression profile of *Zic3-A* and *Zic3–B.* **(A–B)** Semi-quantitative RT-PCR results of *Zic3* isoform specific mRNA expression. **(A)** Developmental expression profile at E10.5, 13 and 16, and **(B)** postnatal tissue specific expression profile. **(C)** Expression of Zic3-A and -B mRNA in HEK293 and P19 cell lines. Approximate band sizes in panels A and C are indicated by arrowheads. Upper  = 300 bp, lower  = 200 bp. cer, cerebellum; mus, skeletal muscle; liv, liver; spl, spleen; brn, brain without cerebellum; hrt, heart; kid, kidney; lng, lung; -RT, control without reverse transcriptase.

In order to confirm the existence of an alternatively-spliced exon *in vivo,* we designed isoform specific primers for murine *Zic3*. Murine embryonic day (e)10.5 RNA served as the template for RT-PCR, and products of the expected sizes for *Zic3-A* and *–B* were amplified and sequenced, confirming the existence of two distinct *Zic3* mRNAs with unique 3′ ends beginning immediately after exon 2. RT-PCR was also used to examine expression of both isoforms at e13 and 16, as well as in a panel of adult tissues (Figure 4A and B). Transcripts of both isoforms of *Zic3* were detected at all three embryonic stages and in adult brain tissue. *Zic3* expression by RT-PCR has been reported in murine heart tissue [30]. Upon extending PCR cycles (45–60) we detected *Zic3-A* expression in murine e10.5 heart tissue, but we did not detect *Zic3-B* expression. Both *Zic3* transcript isoforms were also detected in human embryonic kidney (HEK293) and pluripotent murine teratocarcinoma (P19) cells (Figure 4C).

### Characterization of ZIC3-A and -B proteins

**Figure 5:**
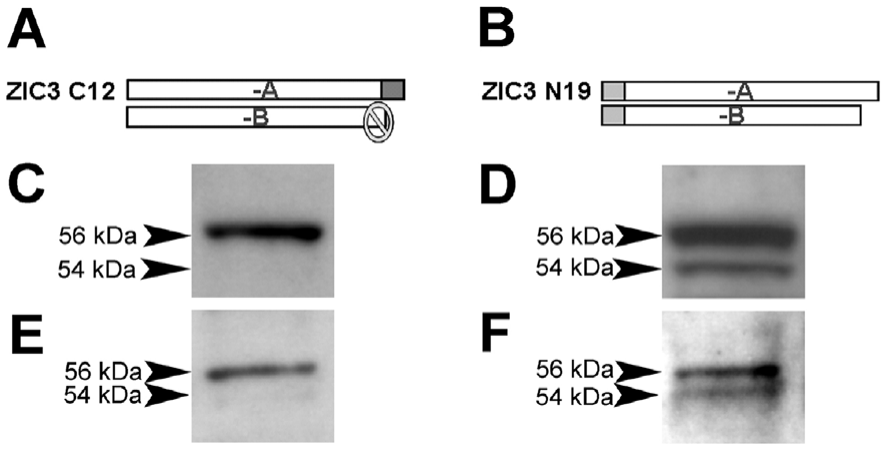
Expression of ZIC3-A and ZIC3-B isoforms in mammalian cells. **(A)** Anti-ZIC3 immunogen specific for C-terminus (C12), and **(B)** for N-terminus (N19). Detection of endogenous ZIC3-A and –B expression in **(C–D)** murine NIH/3T3 and **(E–F)** human fibroblast whole cell lysate using anti-ZIC3 C-terminus **(C, E)** or anti-ZIC3 N-terminus specific antibodies **(D, F)**.

Expression of endogenous ZIC3-A and -B proteins was investigated in both murine and human cell lines. We used ZIC3 specific antibodies that recognize either the C-terminal or N-terminal ends, exploiting the fact that the C-terminal antibody will only recognize ZIC3-A and the N-terminal antibody will recognize both forms (Figure 5 A–B). Using whole cell lysates of NIH/3T3 mouse fibroblasts and Western analysis, we detected a single ZIC3-A band with the C-terminal antibody and two bands with the N-terminal antibody (Figure 5 C–D). Similar results were observed with human fibroblasts (Figure 5 E–F). Taken together with our RNA expression evidence, these data demonstrate the *in vivo* expression of ZIC3-B at both the RNA and protein levels. Therefore we conclude that ZIC3-B is a novel and distinct isoform.

**Figure 6:**
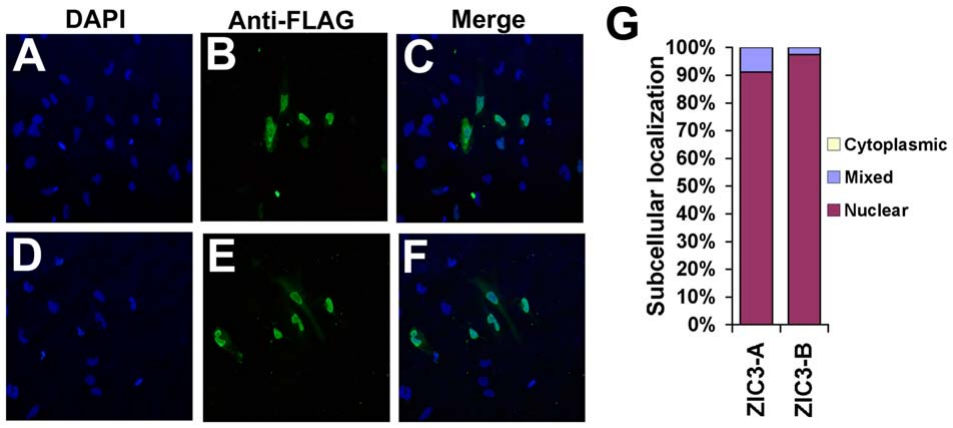
Subcellular localization of ZIC3-A and ZIC3-B. ZIC3 isoform expression in HeLa cells was detected by immunohistochemistry using a mouse anti-FLAG antibody. **(A–C)** Subcellular localization of ZIC3-A. **(D–F)** Subcellular localization of ZIC3-B. **(G)** Quantitative analysis of subcellular localization. Percentages are representative of at least 100 cell counts each from a minimum of six separate experiments.

Previous *in vitro* investigations indicate that ZIC family proteins localize to the nucleus. In order to investigate the subcellular localization of the novel isoform, we performed immunohistochemistry in HeLa cells after transfection with *FLAG-Zic3-B* (Figure 6) and quantified subcellular localization as previously described [4]. Zic3-B is found within the nucleus in approximately 98% of cells (Figure 6G), indicating that this novel isoform is similar to other ZIC family members in its *in vitro* subcellular localization.

### ZIC3-A and –B differ in transcriptional activation ability

ZIC3-A has been previously shown to bind to the Gli consensus binding sequence (GLIBS) *in vitro* by electrophoretic mobility shift assay (EMSA), and it has also been shown to transcriptionally activate luciferase reporters containing multiple, tandem GLIBS [3], [6]. Zhu et al. [3] showed by EMSA that the C-terminal region of ZIC3-A is necessary for binding to the GLIBS and that mutations affecting the C-terminus typically resulted in decreased transcriptional activation of a GLIBS reporter, even when all of the zinc finger domains were intact. As an additional control, a previously described ZIC3-S43X mutant construct was used that encodes only the first 43 amino acids of ZIC3, a region which is identical in both A and B isoforms and which does not contain any zinc finger binding or NLS and NES domains [3], [8], [29]. Because the C-terminal regions of ZIC3-A and –B differ (Figure 2A and C), we postulated that the two isoforms would differ in their ability to activate the 12xGLIBS-luc reporter *in vitro*, either alone or when co-transfected with GLI3.

**Figure 7:**
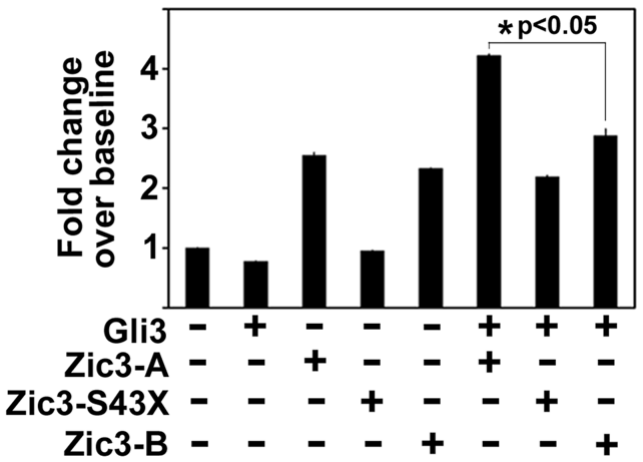
Transcriptional activation mediated by ZIC3 isoforms. NIH/3T3 cells were transfected with Flag-GLI3, HA-ZIC3-A, HA-ZIC3-S43X mutant, or HA-Zic3-B. Transfection of ZIC3-A or Zic3-B alone resulted in similar levels of transcriptional activation above baseline, but GLI3 or ZIC3-S43X alone did not result in activation. Co-transfection of ZIC3-A or ZIC3-S43X with GLI3 resulted in co-activation, but there was no increase in activation when Zic3-B and GLI3 were co-transfected. ZIC3-A acts as a co-activator with GLI3 but Zic3-B does not. (*) Significant difference determined by two-tailed, unpaired Student's t-test assuming unequal variances.

When cells were transfected with ZIC3-A or Zic3-B, low level transcriptional activation was observed compared to baseline activation, and the level of activation of both isoforms was similar (Figure 7). As expected, the ZIC3-S43X mutant was not able to independently activate transcription due to absence of all NLS domains; however, activation was unexpectedly observed upon co-transfection with GLI3. As expected based on previous work [3], increased activation was also seen when ZIC3-A was co-transfected with GLI3 compared to activation with ZIC3-A alone. Interestingly, Zic3-B and GLI3 co-transfections did not result in additional activation compared to Zic3-B alone (Figure 7). Taken together with previous studies, these results indicate that ZIC3-A and GLI3 may be transcriptional co-activators [3], [6]. In contrast, although Zic3-B can induce low level transcriptional activation through the GLIBS alone, it does not co-activate with GLI3.

### Screening a heterotaxy and CHD cohort for mutations in *ZIC3-B*

Although heterotaxy is more common in males, our previous analysis indicated that mutations in ZIC3-A were not responsible for this overrepresentation. To investigate the contribution of mutations in the novel ZIC3-B isoform we performed mutation screening in heterotaxy and CHD cohorts (Table 1). De-identified genomic DNA samples were PCR amplified and sequenced in 109 males, consisting of 5 familial and 104 sporadic cases. Of the 5 familial cases, 2 exhibited a clear X-linked inheritance pattern. No mutations were identified within exon 4. In addition to screening all samples for exon 4, samples which had not been previously analyzed for ZIC3-A mutations were sequenced for these exons as well and no mutations were identified. The mouse model of Zic3 deficiency results in loss of function of both isoforms of Zic3 and has a very severe phenotype with a high percentage of gastrulation defects and early pregnancy loss [2], [12]. Therefore, we cannot rule out the possibility that mutations in exon 4 cause early embryonic lethality or a distinct phenotype not represented in our heterotaxy cohort.

## Discussion

Alternative splicing is a diversification mechanism that allows for increases in proteome complexity via specific regulation of gene expression. Splicing may result in different protein domain combinations, alterations in tissue specificity, or dramatic changes in functional activity, all of which have profound implications for development and disease. Here, we demonstrate that a transcription factor that is important for proper cardiac development exists as two distinct isoforms that are expressed *in vivo* through alternative splicing at the carboxy terminal domain. Pritsker et al. [31] previously demonstrated that splicing is often weakly conserved in orthologous genes in mouse and human. Thus, the conservation of alternatively spliced ZIC3 isoforms in both human and mouse cell lines suggests functional importance.

The ZIC3-B isoform retains most of the mapped domains of ZIC3, including four zinc finger DNA binding domains, two NLS domains, and the NES domain. Thus previously identified patient mutations should be re-evaluated in terms of their functional effect not only on the ZIC3-A isoform, but also on ZIC3-B. Our past findings suggested that the NES domain of ZIC3 is cryptic, and that structural changes, possibly at the carboxy terminus, are required to expose the motif and facilitate export [8]. It will therefore be important to determine the effect of the novel C-terminus identified in ZIC3-B on nuclear export. In addition, the NLS and NES motifs overlap with the zinc fingers of ZIC3, which have previously been shown to bind GLI [32]. GLI transcription factors contain a functionally active bipartite NLS motif as well as an NES, and undergo complex nucleocytoplasmic trafficking in conjunction with SUFU (Suppressor of fused) to mediate hedgehog signaling [23], [33], [34], [35], [36], [37]. Physical interactions between ZIC3 and GLI may result in the coordination of several NLS sites for recruitment to the nucleus and/or alteration in exposure of NES motifs. Studies investigating possible binding partners, including GLI family members, may provide additional clues for further understanding the nucleocytoplasmic shuttling mechanism of ZIC3-A and –B during left-right patterning and cardiac development.

Studies on GLI superfamily members have also shown that post-translational modifications are a primary determinant in the protein's ability to affect transcription [32], [36]. ZIC3 can bind to consensus GLI binding sites to activate transcription, and ZIC3-A appears to act as a transcriptional co-activator in conjunction with GLI3 [3]. The inability of Zic3-B to also act as a transcriptional co-activator with GLI3 may be due to the previously described differences in the fifth zinc finger domain and/or third NLS domain (Figure 2C). More studies are necessary to compare differences in the C-termini of the two isoforms, the subcellular localization of both isoforms and GLI3 proteins when co-transfected, and the effect of potential post-translational modifications of both isoforms [11].

The data indicate that the developmental and tissue specific expression profiles of murine *Zic3-A* and *Zic3-B* overlap. Previous studies have shown that alternatively spliced isoforms that are co-expressed may act as negative regulators [38]. It will be important to examine the co-regulation of the –A and –B isoforms during development. Our initial mutation analyses did not identify pathogenic mutations in exon 4. Because of the small number of families available for study, analysis of additional X-linked pedigrees will be required to establish the prevalence of the alternatively spliced isoform in familial cases. Given that ZIC3-A mutations have been identified at relatively low rates (approximately 1%) in sporadic heterotaxy cases and isolated congenital heart defects, analysis of exon 4 of the ZIC3-B isoform in larger cohorts is important to more clearly establish its contribution to disease.

## References

[1] Clark KL, Yutzey KE, Benson DW (2006). Transcription factors and congenital heart defects.. Annu Rev Physiol.

[2] Purandare SM, Ware SM, Kwan KM, Gebbia M, Bassi MT (2002). A complex syndrome of left-right axis, central nervous system and axial skeleton defects in Zic3 mutant mice.. Development.

[3] Zhu L, Zhou G, Poole S, Belmont JW (2007). Characterization of the Interactions of Human ZIC3 Mutants With GLI3.. Hum Mut.

[4] Aruga J, Yokota N, Hashimoto M, Furuichi T, Fukuda M (1994). A novel zinc finger protein, zic, is involved in neurogenesis, especially in the cell lineage of cerebellar granule cells.. J Neurochem.

[5] Pavletich NP, Pabo CO (1993). Crystal structure of a five-finger GLI-DNA complex: new perspectives on zinc fingers.. Science.

[6] Mizugishi K, Aruga J, Nakata K, Mikoshiba K (2001). Molecular properties of Zic proteins as transcriptional regulators and their relationship to GLI proteins.. J Biol Chem.

[7] Lim LS, Hong FH, Kunarso G, Stanton LW (2010). The Pluripotency Regulator Zic3 is A Direct Activator of the Nanog Promoter in Embryonic Stem Cells.. Stem Cells.

[8] Bedard JE, Purnell JD, Ware SM (2007). Nuclear import and export signals are essential for proper cellular trafficking and function of ZIC3.. Hum Mol Gen.

[9] Zhu L, Zhou G, Poole S, Belmont JW (2007). Characterization of the Interactions of Human ZIC3 Mutants With GLI3.. Human Mutation.

[10] Ware SM, Peng J, Zhu L, Fernbach S, Colicos S (2004). Identification and functional analysis of ZIC3 mutations in heterotaxy and related congenital heart defects.. Am J Hum Genet.

[11] Ware SM, Harutyunyan KG, Belmont JW (2006). Zic3 is critical for early embryonic patterning during gastrulation.. Dev Dyn.

[12] Ware SM, Harutyunyan KG, Belmont JW (2006). Heart defects in X-linked heterotaxy: evidence for a genetic interaction of Zic3 with the nodal signaling pathway.. Dev Dyn.

[13] Nakata K, Nagai T, Aruga J, Mikoshiba K (1997). Xenopus Zic3, a primary regulator both in neural and neural crest development.. Proc Natl Acad Sci USA.

[14] Nagai T, Aruga J, Takada S, Gunther T, Sporle R (1997). The expression of the mouse Zic1, Zic2, and Zic3 gene suggests an essential role for Zic genes in body pattern formation.. Dev Biol.

[15] Aruga J, Nagai T, Tokuyama T, Hayashizaki Y, Okazaki Y (1996). The mouse zic gene family. Homologues of the Drosophila pair-rule gene odd-paired.. J Biol Chem.

[16] Tzschach A, Hoeltzenbein M, Hoffmann K, Menzel C, Beyer A (2006). Heterotaxy and cardiac defect in a girl with chromosome translocation t(X;1)(q26;p13.1) and involvement of ZIC3.. Eur J Hum Genet.

[17] Megarbane A, Salem N, Stephan E, Ashoush R, Lenoir D (2000). X-linked transposition of the great arteries and incomplete penetrance among males with a nonsense mutation in ZIC3.. Eur J Hum Gen.

[18] Kitaguchi T, Nagai T, Nakata K, Aruga J, Mikoshiba K (2000). Zic3 is involved in the left-right specification of the Xenopus embryo.. Development.

[19] Gebbia M, Ferrero GB, Pilia G, Bassi MT, Aylsworth A (1997). X-linked situs abnormalities result from mutations in ZIC3.. Nat Genet.

[20] De Luca A, Sarkozy A, Consoli F, Ferese R, Guida V (2010). Familial transposition of the great arteries caused by multiple mutations in laterality genes.. Heart.

[21] Czosek RJ, Haaning A, Ware SM (2010). A mouse model of conduction system patterning abnormalities in heterotaxy syndrome.. Ped Res.

[22] Johnson JM, Castle J, Garrett-Engele P, Kan Z, Loerch PM (2003). Genome-wide survey of human alternative pre-mRNA splicing with exon junction microarrays.. Science.

[23] Kogerman P, Grimm T, Kogerman L, Krause D, Unden AB (1999). Mammalian suppressor-of-fused modulates nuclear-cytoplasmic shuttling of Gli-1.. Nat Cell Biol.

[24] Koyabu Y, Nakata K, Mizugishi K, Aruga J, Mikoshiba K (2001). Physical and functional interactions between Zic and Gli proteins.. J Biol Chem.

[25] Dai P, Akimaru H, Tanaka Y, Maekawa T, Nakafuku M (1999). Sonic Hedgehog-induced Activation of the Gli1Promoter Is Mediated by GLI3.. J Biol Chem.

[26] Lowry OH, Rosebrough NJ, Farr AL, Randall RJ (1951). Protein measurement with the Folin phenol reagent.. J Biol Chem.

[27] Frazer KA, Pachter L, Poliakov A, Rubin EM, Dubchak I (2004). VISTA: computational tools for comparative genomics.. Nucleic Acids Res.

[28] Sakai-Kato K, Ishiguro A, Mikoshiba K, Aruga J, Utsunomiya-Tate N (2008). CD spectra show the relational style between Zic-, Gli-, Glis-zinc finger protein and DNA.. Biochimica et Biophysica Acta.

[29] Hatayama M, Tomizawa T, Sakai-Kato K, Bouvagnet P, Kose S (2008). Functional and structural basis of the nuclear localization signal in the ZIC3 zinc finger domain.. Hum Mol Genet.

[30] Zhu L, Harutyunyan KG, Peng JL, Wang J, Schwartz RJ (2007). Identification of a novel role of ZIC3 in regulating cardiac development.. Mol Genet.

[31] Pritsker M, Doniger TT, Kramer LC, Westcot SE, Lemischka IR (2005). Diversification of stem cell molecular repertoire by alternative splicing.. Proc Natl Acad Sci U S A.

[32] Sheng T, Chi S, Zhang X, Xie J (2006). Regulation of Gli1 localization by the cAMP/protein kinase A signaling axis through a site near the nuclear localization signal.. J Biol Chem.

[33] Stone DM, Murone M, Luoh S, Ye W, Armanini MP (1999). Characterization of the human suppressor of fused, a negative regulator of the zinc-finger transcription factor Gli.. J Cell Sci.

[34] Ding Q, Fukami S, Meng X, Nishizaki Y, Zhang X (1999). Mouse suppressor of fused is a negative regulator of sonic hedgehog signaling and alters the subcellular distribution of Gli1.. Curr Biol.

[35] Barnfield PC, Zhang X, Thanabalasingham V, Yoshida M, Hui CC (2005). Negative regulation of Gli1 and Gli2 activator function by Suppressor of fused through multiple mechanisms.. Differentiation.

[36] Pan Y, Bai CB, Joyner AL, Wang B (2006). Sonic hedgehog signaling regulates Gli2 transcriptional activity by suppressing its processing and degradation.. Mol Cell Biol.

[37] Dunaeva M, Michelson P, Kogerman P, Toftgard R (2003). Characterization of the physical interaction of Gli proteins with SUFU proteins.. J Biol Chem.

[38] Kuiper RP, Schepens M, Thijssen J, Schoenmakers EF, van Kessel AG (2004). Regulation of the MiTF/TFE bHLH-LZ transcription factors through restricted spatial expression and alternative splicing of functional domains.. Nucleic Acids Res.

